# The roles and mechanisms of epigenetic regulation in pathological myocardial remodeling

**DOI:** 10.3389/fcvm.2022.952949

**Published:** 2022-08-26

**Authors:** Kun Zhao, Yukang Mao, Yansong Li, Chuanxi Yang, Kai Wang, Jing Zhang

**Affiliations:** ^1^Department of Cardiology, The First Affiliated Hospital of Nanjing Medical University, Nanjing, China; ^2^Department of Cardiology, Yangpu Hospital, Tongji University School of Medicine, Shanghai, China

**Keywords:** epigenetic regulation, pathological myocardial remodeling, DNA methylation, histone posttranslational modification, adenosine disodium triphosphate (ATP)-dependent chromatin remodeling, RNA modification

## Abstract

Pathological myocardial remodeling was still one of the leading causes of death worldwide with an unmet therapeutic need. A growing number of researchers have addressed the role of epigenome changes in cardiovascular diseases, paving the way for the clinical application of novel cardiovascular-related epigenetic targets in the future. In this review, we summarized the emerged advances of epigenetic regulation, including DNA methylation, Histone posttranslational modification, Adenosine disodium triphosphate (ATP)-dependent chromatin remodeling, Non-coding RNA, and RNA modification, in pathological myocardial remodeling. Also, we provided an overview of the mechanisms that potentially involve the participation of these epigenetic regulation.

## Introduction

Pathological myocardial remodeling, characterized as cardiomyocyte hypertrophy, apoptosis, and excessive fibrosis, is an adaptive growth process that occurs in response to the capacity/pressure load caused by certain diseases, environmental factors, or lifestyles (such as long-term hypertension, atherosclerosis, myocardial infarction, metabolic abnormalities, smoking, drinking, etc.), which ultimately leads to a decrease in heart output and an increased risk of heart failure (1). Thus, it is of great interest to develop novel therapeutic approaches targeting the mechanisms underlying the progression of chronic heart failure-induced pathological myocardial remodeling.

**Figure 1:**
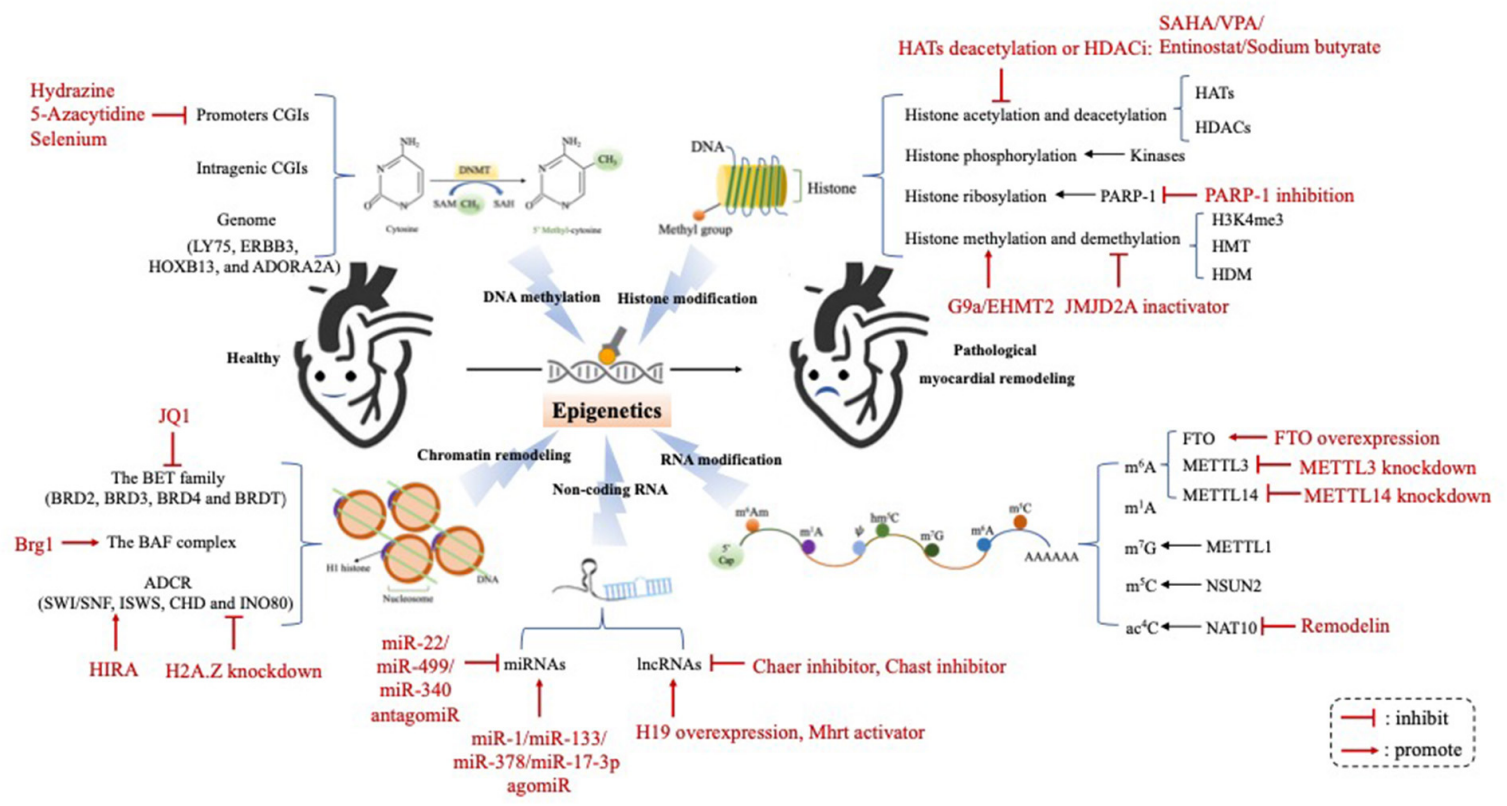
The epigenetic mechanism underlying the occurrence and progression of pathological myocardial remodeling. Epigenetic regulation underlying the development of pathological myocardial remodeling can be divided into four different molecular levels: (1) DNA methylation; (2) Histone modification; (3) Chromatin remodeling; (4) Non-coding RNA; (5) RNA modification. HATs, histone acetyltransferases; HDACs, histone deacetylases; PARP-1, poly (ADP-ribose) polymerase-1; H3K4me3, histone 3 lysine 4 triamcinolone; HMT, histone methyl transferase; HDM, histone trimethyldemethylase; FTO, Fat mass and obesity-associated protein; METTL3, methyltransferase-like 3; METTL14, methyltransferase-like 14; m^6^A, N^6^-methyladenosine; m^1^A, N^1^-methyladenosine; m^7^G, N^7^-methylguanosine; m^5^C, 5-methylcytosine; ac4C, N^4^-acetylcytidine; miRNAs, microRNAs; lncRNAs, long ncRNAs; Chast, cardiac hypertrophy-associated transcript; Chaer, cardiac-hypertrophy-associated epigenetic regulator.

The term “Epigenetic” is a combination of the Greek prefix “epi” (meaning “up,” or “around”), and “genetics.” Thus, epigenetics can be defined as the heritable regulation of gene expression by modifying chromosomal components without altering the genome nucleotide sequence (2). Epigenetic regulation can be divided into five different molecular levels: (1) DNA methylation; (2) Histone posttranslational modification; (3) Adenosine disodium triphosphate (ATP)-dependent chromatin remodeling; (4) Non-coding RNA; (5) RNA modification (3). It maintains cellular characteristics and controls cell differentiation by regulating chromatin structure and gene expression at various molecular levels, which is essential for normal development and disease progression. A growing body of research has shown that epigenome changes may be the markers of various cancers, and several molecules have been identified as the potential targets for some novel therapeutics (4). Notably, recent genetic and biochemical analyses suggested that epigenetic changes play an important role in the occurrence and progression of myocardial remodeling. In this review, we will summarize recent advances, and provide an overview of epigenetic studies targeting pathological myocardial remodeling in human and animal models (Figure 1).

## DNA methylation

DNA methylation in eukaryotic organisms occurs primarily at the 5th carbon atom (5 mC) of the cytosine ring, followed by guanine dinucleotides, where cytosine and guanine are separated by phosphate (CpG). DNA methylation plays a vital role in gene regulation, especially in transcriptional inhibition depending on methylation location. Increased methylation in CpG-enriched regions (CpG islands (CGI) in the gene promoter region) is commonly associated with gene silencing, whereas methylated CpG found in the genome is associated with gene activation (5).

The altered DNA methylation landscape were initially known as critical epidemiological markers underpinning various human diseases, especially in the process of carcinogenesis (6). During the past decade, extensive genome-wide studies have provided robust evidence that DNA methylation is closely involved in the pathogenesis of myocardial remodeling associated with different etiologies, including dilated cardiomyopathy (DCM), ischemic cardiomyopathy (ICM), chronic pressure overload and valve disease (7–11). Movassagh et al. demonstrated for the first time that a huge amount of CGI and promoters are hypomethylated at the end stages of heart failure (7). The previous study determined that the differential DNA methylation in failing and non-failing human myocardium did not occur uniformly throughout the genome using methylated DNA immunoprecipitation (MeDIP) (8).

Recent study found that the overall CpG methylation was increased in DCM hearts compared with the control hearts (12). In 2013, Haas et al. detected different patterns of DNA methylation in left ventricular tissues obtained from DCM patients and reproduced epigenetic regulation patterns of several genes with previously unknown functions in DCM, including lymphocyte antigen 75 (LY75), tyrosine kinase-type cell surface receptor HER3 (ERBB3), Homeobox B13 (HOXB13), and adenosine receptor A2A (ADORA2A) (13). Then the functional correlation of these identified genes in the adaptive or maladaptive pathways in heart failure was further demonstrated in zebrafish (13), suggesting that it is reasonable to investigate their potential as diagnostic and therapeutic targets specifically in DCM but also in heart failure.

Genome-widely DNA methylation analysis in combination with RNA sequencing revealed a robust gene expression pattern that is consistent with suppression of oxidative metabolism, induced anaerobic glycolysis, and deteriorated myocardial remodeling in the ischemic hearts of patients with end-stage heart failure in comparison with non-ischemic control hearts, with the Krüppel-like factor 15 (KLF15) and polycomb methyltransferase enhancer of zeste 2 polycomb repressive complex 2 subunit (EZH2) being identified as candidate regulators of ICM-associated epigenetic reprogramming (9), indicating that these genes may be relevant druggable targets in myocardial remodeling subsequent to heart failure.

## Histone posttranslational modification

Histones are part of the chromatin complex that forms chromosomes. The histone octamer contains 2 copies of the H2A, H2B, H3, and H4 proteins, forming the core of the nucleosome, which is wrapped 1.67 times by approximately 147 base pairs (14). Unlike DNA methylation, the role of histone modifications in epigenetic regulations appear to be more subtle. The function of histone modification depends on not only the position, but also the type and amount of histone modification. Histone posttranslational modifications include acetylation, methylation, phosphorylation, ribosylation, etc. at different amino acid residues, which may alter the chromatin structure and/or influence the downstream genes expression via recruiting different kinds of regulatory molecules (e.g., histone modifiers, chromatin regulators, and transcription factors) (15, 16). Although it is difficult to decode specific posttranslational modifications at the level of individual histone and mononucleosome, a growing body of evidence has demonstrated that histone modifications may interact to exert transcriptional regulatory effects (17).

### Histone acetylation and deacetylation

Histone acetylation and deacetylation acetylation/deacetylation are the most common histone modifications, which are catalyzed by histone acetyltransferases (HATs) and histone deacetylases (HDACs), respectively (18). Findings from previous studies performed on mammalian cells have determined a strong association of histone acetylation and deacetylation with numerous human diseases, including cancer (19), neurological disorders (20) and cardiovascular disease (CVD) (21). Histone acetylation of HATs may disrupt the interactions within and between nucleosomes, thereby “relaxing” chromatin structures and activating transcription. In contrast, HDACs deacetylate histones, which increased histone-DNA interactions, leading to chromatin concentration and gene inhibition (22). It has been demonstrated that specific HATs and HDACs are critical modulators of cardiac hypertrophic response and contribute to the development of pathological myocardial remodeling (21).

In 2007, Montgomery et al. showed that the loss of HDAC1 led to the death of mouse embryos on Day 9.5, but mice lacking HDAC2 survived to the perinatal period and eventually died of heart defects, such as right ventricular cavity occlusion, myocardial cell apoptosis, and bradycardia (23). Besides, cardiac-specific deletion of HDAC1 or HDAC2 resulted in neonatal death, with arrhythmia, or DCM (23).

Also, Trivedi et al. found that the transgenic mice overexpressed with HDAC2 developed exaggerated myocardial hypertrophy (24). In contrast to HDAC2, cardiomyocyte-specific overexpression of HDAC3 in mice increased cardiomyocyte proliferation, rather than promoting cardiac hypertrophy (25). In 2008, Montgomery et al. showed that cardiac-specific HDAC3-deficient mice survived up to 3–4 months after birth, presenting with severe myocardial hypertrophy (26). Moreover, some researchers have found that HDAC4, HDAC5, HDAC7, and HDAC9 can attenuate cardiac hypertrophy through binding to transcriptional factor myocyte enhancer factor 2 (MEF2) and suppressing its activity, supporting the role of these endogenous factors in inhibiting pathological cardiac growth and preserving cardiac function (27–29). On this basis, Helmstadter et al. further demonstrated that calcium/calmodulin-dependent protein kinase II (CaMKII) and protein kinase A (PKA)-dependent phosphorylation co-regulated the nuclear localization of HDAC4 in adult cardiomyocytes, which has significant transcriptional implications in the development of heart failure (30).

In addition, CREB binding protein (CBP)/p300 may drive unrestricted cardiac growth and impaired cardiac geometry by acetylating and increasing the transcriptional activity of MEF2 (31). Cardiac-specific overexpression of p300 in mice increases GATA4 acetylation, resulting in significant centrifugal dilation and contraction dysfunction of the heart (32). In contrast, Oral curcumin (a p300-specific HAT inhibitor) administration was reported to prevent the deterioration of cardiac function in salt-sensitive Dahl rats and surgically induced myocardial infarction (MI) rats (33). Therefore, the deacetylation or inhibition of HATs may be beneficial in ameliorating pathological myocardial remodeling in patients with heart failure.

The pioneering studies demonstrated that the homologs of Silent information regulator 2 (Sir2) (i.e., Sirtuins 1–Sirtuins 7), a family of histone deacetylases located in different cellular compartments whose function depends on nicotinamide adenine dinucleotide (NAD+), play a non-negligible physiopathological role in regulating energy metabolism, senescence, and other biological processes under various pathological cellular processes via acting on different targets in a non-redundant manner (34, 35). The knockdown of Sirtuins may result in epigenetic silencing and defected DNA repair process, leading to shorter lifespan of yeast Saccharomyces cerevisiae and different cardiovascular diseases in mammals (36, 37).

Among the family of Sirtuins, Sirtuins 1 (SIRT1) is the most well-studied one since it is the closest homolog of Sir2 (38). SIRT1 was found to be involved in regulating DNA repair, gene transcription, oxidative stress, and longevity (39, 40). The enhanced activity of SIRT1 was depicted to exert a promising cardioprotective effects against arteriosclerotic cardiovascular disease (ASCVD) by deacetylating the DNA repair machinery components (38, 39).

Interestingly, SIRT1 expression which decreases with aging correlated with the onset and development of heart failure and even ST-segment elevation myocardial infarction (STEMI) (41–43). Heart failure-induced miRNA-138-5p expression may downregulate the activity of SIRT1, thereby enhancing the acetylation of p53, which triggers cardiomyocyte apoptosis (44). Also, pharmacological activation of SIRT1 could improve cardiac contractile dysfunction by inhibiting the acetylation of SERCA2a at K492 (45). Besides, the *in-vitro* experiments found that sodium-dependent glucose transporters 2 (SGLT2) inhibitors may play a cardioprotective role under hyperglycemia conditions through activating SIRT1/PGC-1α/FGF21 signaling pathway (46).

Sirtuins 3 (SIRT3), a deacetylase which may regulate mitochondrial function, also plays a cardioprotective effects against energy dyshomeostasis and cardiac dysfunction (47). Like SIRT1, the declined activity of SIRT3 associated with aging is associated with endothelial dysfunction, hypertension, and heart failure (48). Emerging evidence demonstrated that the stimulated SIRT3 activity could ameliorate cell death, fibrosis, and the inflammatory response of cardiomyocytes under the hypertrophic stimulus, thus improving health and lifespan (49–51). Besides, silencing SIRT3 may sensitize overload-induced heart failure by worsening cardiomyocyte glucose utilization (52), whereas the impaired SIRT3-mediated endothelium cell metabolism could even result in cardiac diastolic dysfunction (53).

### Histone methylation and demethylation

In 1964, histone methylation was first described by Murray (54). The first evidence linking histone methylation to transcription was presented in 1999 by reporting that histone H3 arginine-specific methyltransferase CARM1 interacted with the steroid hormone receptor co-activator GRIP-1 during transcription initiation (55).

The activation or inhibition of histone methylation to genes depends on which residue occurs on histone. For example, histone 3 lysine 4 triamcinolone (H3K4me3) is associated with active promoter activity, whereas trimethylation of H3K9me3 is usually related to transcription inhibition (15, 56).

During heart failure, H3K4me3 increased in the promoter and transcription region of myosin heavy chain (β-MHC), while H3K4me3 decreased at the α-MHC promoter (57), suggesting that “classic” transcription reprogramming that occurred during myocardial remodeling involved not only the reactivation of the fetal heart genes, but also the changes of methylation patterns at the same gene site that are reactivated under pathological stress.

It has been shown that histone methyl transferase (HMT), G9a/EHMT2, was considered essential for homeostasis of cardiomyocytes in the adult heart (58). The study found that conditional abnormalities of G9a silencing in 2-month-old mouse myocardium caused various cardiac dysfunctions in the first 4 weeks of knockout induction. G9a, which inhibited the key cardiac regulatory genes through H3K9me2, was upregulated during the initial stages of heart failure. In addition, the chemical inactivation of G9a in mice undergoing aortic narrowing surgery (TAC) improved cardiac function and inhibited the development of remodeling (58). Thus, G9a acted as an anti-hypertrophy regulator in healthy hearts, and a hypertrophy activator in stress-induced hearts.

Zhang et al. demonstrated that histone trimethyldemethylase (HDM), JMJD2A, promoted myocardial remodeling in TAC-induced mice (59). After TAC surgery, JMJD2A bound to the promoter regions of Four and a Half LIM Domains Protein 1 (FHL1), and reduced H3K9me3 levels in the heart, which may account for the ameliorative effects of JMJD2A inactivation on myocardial remodeling.

Finally, mis-regulation of histone methylation cofactors also affected myocardial remodeling. Specific deletion of the PAX transcriptional activation domain interaction protein (PTIP), a cofactor of H3K4 methylation, in adult mice resulted in a reduction in H3K4me3 (60).

### Histone phosphorylation

Protein kinases deliver extracellular signals from the cell surface to the nucleus. Kinases could not only phosphorylate cellular proteins, and transcription factors, but also regulate major cellular processes such as transcription, mitosis, DNA damage, and apoptosis via mediating chromatin dynamics. Although histones can be phosphorylated by stress signals, most studies still focused on the role of histone acetylation and methylation in controlling transcription of cardiac genes. Thus, histone phosphorylation was poorly studied and understood. Since many kinases are key mediators of myocardial remodeling, however, whether they acted as physiological histone kinases to regulate transcription remains debatable (61).

### Histone ribosylation

Histone ribosylation is mediated by poly (ADP-ribose) polymerase-1 (PARP-1), which transfers the ADP-ribose group from nicotinamide adenine dinucleotide (NAD +) to the receptor substrate (62). Substrates for PARP-1 include PARP-1 itself and the tails of histones H1, H2A, H2B, H3, and H4 (63). Histone ribosylation could induce relaxation of chromatin structure, which helps recruit repair enzymes to damaged DNA sites. In addition to playing a role in DNA repair, PARP also regulates cell death, cell cycle progression, and genomic stability (64). In myocardium, PARP-1 was reported to promote myocardial remodeling, and increased PARP-1 activity led to heart failure in mice and humans (65). In contrast, PARP-KO mice were resistant to angiotensin II-induced myocardial remodeling (65, 66). The mechanism of PARP-1 inducing pathological hypertrophy was achieved by interacting with HDAC and the chromatin remodeler BRG-1 (67). In addition to its effects on fetal cardiac genes, PARP also controlled cardiac hyperplasia via regulating the P38 MAP kinase, ERK1/2, PI3 kinase-Akt-GSK3β, and JNK signaling pathways (68, 69).

### Genome-wide study of histone posttranslational modification

At present, several genome-wide studies of myocardial histone posttranslational modifications have been performed in animal models or humans. In 2009, Kaneda et al. published the first genome-wide study of myocardial histone methylation in humans and rodents, showing the significant differences in the distribution patterns of H3K4me3 and H3K9me3 between healthy and failing hearts (70). In 2011, Movassagh et al. also demonstrated that H3K36me3 showed different methylation patterns and enrichment between normal human myocardium and cardiomyopathy hearts (7). In 2013, Papait et al. analyzed seven histone epigenetic markers in the heart tissues of control and TAC mice by ChIP sequencing, which found that histone acetylation levels (e.g., H3K9ac and H3K27ac) were reduced around down-regulated gene loci in TAC mice (71). Besides, the more active the transcription of differentially expressed genes in TAC mice, the fewer histone inhibitory markers (e.g., H3K27me3, H3K9me2, and H3K9me3) they observed.

In 2018, Gilsbach et al. used nuclear staining and fluorescence-assisted sorting to isolate human myocardial cells from normal fetuses, infants, and adults' normal and failing hearts for genome-wide epigenetics (72). The results showed that the whole-transcriptomic regulation of normally methylated cells during normal development was determined mainly by the histones commonly observed on cis-acting elements at the distal end of the genome. Another interesting finding of this study was that the expression of genes associated with heart failure was regulated by activity-enhanced histone markers, but not by DNA methylation.

## ATP-dependent chromatin remodeling

The dynamic epigenome modifications, including histone posttranslational modification and DNA methylation, may trigger chromatin remodeling, which is considered an important process for controlling cell fate and determining organ development (73). Chromatin regulators, such as histone modifiers, cofactors, and transcription regulators, could recruit other chromatin regulators and/or transcription factors to regulate genes expression. By collecting all the necessary regulators, the distal cis-acting elements (such as enhancers) could form chromatin rings that interact with the proximal promoter region, thereby initiating and enhancing the transcriptional activity of the genes with underlying transcription complexes (73).

Studies regulating the function of bromine domain (BET) proteins, acetylate lysine binding proteins and epigenetic readers have shown that chromatin structures were critical for regulating the dynamics of gene expression in the failed heart (74, 75). The BCD domain (BET) family includes BRD2, BRD3, BRD4, and BRDT. Spiltoir et al. found that hypertrophic stimulation promoted recruitment of BRD4 to the transcription start site (TSS) of the gene encoding atrial natriuretic factors (ANF) (74).

In addition to the histones and coactivators mentioned above, the ATP-dependent chromatin remodeling complex (ADCR) is another regulator that may play a key role in chromatin remodeling (76). ADCR contains a subunit of adenosine triphosphate (ATPase) belonging to the SNF2 protein superfamily, which uses ATP hydrolysis as an energy source to alter or disrupt Histone-DNA interactions. In mammals, four ADCR families (SWI/SNF, ISWS, CHD and INO80) have been identified (77). Among them, the role of SWI/SNF in cardiac development and remodeling has been extensively studied over the past decade (78–82). These studies found that the BAF complex (the mammalian analog of the SWI/SNF complex) was generally abundant in the embryonic heart but down-regulated in the adult myocardium. Myosin heavy chain (MHC) is an important molecule that ensures the normal operation of the heart. There are two main subtypes, namely α-MHC and β-MHC, which are expressed specifically in the mammalian heart and located on the same chromosome (83). α-MHC, which has higher ATPase activity than β-MHC, is mainly expressed in adult cardiomyocytes, while β-MHC is expressed in embryonic cardiomyocytes.

How does the BAF complex regulate the expression of these two homogenous types? Based on the study of the mouse model of myocardial-specific ablation of BRG1 (a nucleosome remodeling factor) at different stages of development, it was found that BRG1 was an important ATPase subunit of the BAF complex. BRG1 could interact with HDAC and PARP-1, thereby inhibiting the α-MHC of the adult hearts, and activating the β-MHC of the embryonic hearts. Since the fact that the BAF complex was down-regulated in adult cardiomyocytes, the inhibition of α-MHC was eliminated as well. Thus, MHC (α-MHC) expression was upregulated in the adult hearts (67). In hypertrophic and failing hearts, subunits of the BAF complex and their binding partners, as well as the fetal MHC, were increased (67).

In addition, the effects of BRG1 on recruiting histone methyltransferase G9a/DNA methyltransferase (DMNT3) complex to the promoter regions of Myh6 gene encoding α-MHC was reported to lead to the deposition of H3K9me3, and CpG methylation in the Myh6 promoter regions, which may subsequently activate the inhibitory mechanism of the Myh6 gene in the adult failing hearts (84). Besides, cardiac myocyte-specific ablation of BRG1 attenuated myocardial remodeling in TAC-induced mice. Thus, BRG1 may act as a stress-activated chromatin remodeler that controls fetal reprogramming of cardiac genes (67).

Previous report demonstrated that SWI/SNF chromatin remodeling complex SWR1 could exchange H2A with H2A.Z at specific genomic loci to maintain genomic integrity and facilitate transcriptional initiation (85). In spit to the upregulated expression of the histone variant H2A.Z in the hearts of TAC mice, shRNA knockdown of H2A.Z was also reported to attenuate myocardial remodeling and down-regulate expression of growth-related genes, such as cyclin-dependent kinase 7 and ribosomal S6 in cultured rat cardiomyocytes (86). In addition, cardiomyocyte-specific conditional knockout of the histone chaperone HIRA in mice induced pathological myocardial remodeling, as well as the impaired intramuscular integrity (87). The above studies showed that ATP-dependent chromatin remodeling may play a crucial role in the pathogenesis of myocardial remodeling under different pathological stimuli.

## Non-coding RNA

Protein-coding genes account for a tiny percentage of (3%) of the human genome (88). Recent advances in transcriptomics and bioinformatics have revealed that, remaining 97% untranslated RNAs, defined as non-coding RNAs (ncRNAs), plays a critical role at transcriptional and translational level in both physiological and pathological conditions. Apart from some traditional and well-established ncRNAs, including transfer RNAs (tRNAs) and ribosomal RNAs (rRNAs), the other unidentified ncRNAs can be classified into two major categories based on their sizes, namely, short ncRNAs, which comprises fewer than 200 nucleotides and mainly includes microRNAs (miRNAs), small interfering RNA (siRNAs), small nuclear RNAs (snRNAs), small nucleolar RNAs (snoRNAs), small cytoplasmic RNAs (scRNAs), extracellular RNA (exRNAs), piwi-interacting RNAs (piRNAs), and long ncRNAs (lncRNAs) consisting of more than 200 nucleotides (89).

MicroRNAs (miRNAs) can be defined as short ncRNAs ranging 18–25 nucleotides in length, which are emerging as key regulators involved in the development and progression of CVDs, predominantly due to inhibiting translation or degrading target messenger RNAs (mRNAs) (90). A large amount of miRNAs appear to be differentially expressed between hypertrophic and non-hypertrophic cardiac tissues and regulate different cell types and pathways during myocardial remodeling. Sayed et al. found that miR-1 was downregulated at an early stage in a mouse model of myocardial remodeling induced by transverse aortic constriction (TAC), and that overexpression of miR-1 in cultured cardiomyocytes decreased the levels of growth-related factors including Ras GTPase-activating protein (RasGAP), cyclin-dependent kinase 9 (Cdk9), Ras homolog enriched in brain (Rheb) and fibronectin (91). Similarly, miR-133 expression level has been shown to be significantly augmented in human and murine models of myocardial remodeling. Both *in vitro* and *in vivo* functional studies indicated that miR-133 silence induced by adenoviral transfection or antagomiR led to exacerbated myocardial remodeling and impaired cardiac function, while its overexpression showed opposite effects, with Rhoa, Cdc42, and NELFA/Whsc2 being identified as target genes of miR-133 by bioinformatics analysis (92). Also, miR-21 overexpression was reported to trigger cardiac fibroblasts activation and promote cardiomyocytes proliferation against stress overloaded conditions (93). In PE- or AngII-induced cardiomyocyte hypertrophic model, miR-22 is the most remarkably upregulated miRNA, increases the cell size and regulates the fetal gene expression (nppa and myh6), with PTEN acting as its potential downstream target (94). Cai et al. indicated that miR-765 expression was enhanced in human falling hearts, and that overexpression of miR-765 resulted in reduced inhibitor-1 expression, which subsequently suppressed cardiomyocyte contractile function and calcium cycling through regulation of the PP-1 signaling axis (95). miR-499 levels were increased in human and mouse failing hearts after chronic pressure overload, and its upregulation adversely affect the cardiac function and geometry (96). miR-340 is a pro-eccentric hypertrophy miRNA, which plays a pivotal role, at least in part though targeting cardiomyocyte structure protein DMD, in the development of myocardial remodeling and heart failure associated with DCM, and whose expression can be largely attributed to cytokine CT-1 activation (97). Interestingly, some miRNAs have been documented to stimulate cardiomyocyte proliferation, and thus may function as promising therapeutic targets in cardiac regeneration. Xiao et al. and Shi et al. have demonstrated that miR-31 and miR-17 promotes postnatal cardiomyocyte proliferation and cardiac repair after ischemia-reperfusion injury, with RhoBTB1 and TIMP3 being the main target gene, respectively (98, 99).

Recently, a novelty research identified that Angiotensin receptor/Neprilysin inhibitor (ARNI) may improve maladaptive myocardial remodeling in cardiac resynchronization therapy with defibrillator (CRTd) non-responder patients via modulating miRNAs including miR-18, miR-145, and miR-181 (100).

The mechanisms by which lncRNAs regulate gene expression seem to be complex and multifaceted, which may involve chromatin modification, transcription, post-transcriptional processing and affecting miRNA-mediated gene regulation (101). Kumarswamy and co-workers reported that the level of LIPCAR, a mitochondria-derived lncRNA, was markedly upregulated in cardiac tissues from patients with left ventricular remodeling and chronic heart failure, and that high LIPCAR levels were strongly associated with a higher risk of cardiovascular death (102). RNA sequencing analysis revealed a distinct cardiac ncRNAs expression profile in which lncRNA H19 and its encoded miR-675 comprised the most dominantly upregulated ncRNAs since the early phase after TAC-induced myocardial remodeling in mice. Adenovirus-mediated overexpression and a siRNA-mediated silencing of H19 suppressed hypertrophic growth in neonatal cardiomyocytes, whereas knockdown of H19 accentuated myocardial remodeling (103). Moreover, Wang et al. identified a heart-enriched lncRNA, termed as cardiac-hypertrophy-associated epigenetic regulator (Chaer), which plays a crucial role in the development of myocardial remodeling via interacting with catalytic subunit of polycomb repressor complex 2 (PRC2). The interactions between Chaer and PRC2 inhibits histone H3 lysine 27 methylation (H3K27me3) at the promoter regions of genes, thus attenuating myocardial remodeling in human induced pluripotent stem cell-derived cardiomyocytes (hiPSC-CMs), neonatal rat cardiomyocytes and mouse model after TAC surgery (104).

A new cluster of lncRNA transcripts, namely myosin heavy-chain-associated RNA transcripts (Mhrt), has been found to be derived from a two-exon gene located on Myh7 loci by Han et al. with Mhrt779 being the most abundant species, and characterized by high cardiac-specificity, high abundance in the adult heart and potent cardioprotective effects. To be noted, a diminished Mhrt expression was observed in TAC-induced hypertrophic mouse hearts, which may be partially attributed to Brg1-Hdac-Parp complex. Collectively, these findings provide novel insights into the regulatory roles of Mhrt in myocardial remodeling, implying the property for Mhrt as a promising therapeutic target in pathological cardiac remodeling (105).

Using whole-genome sequencing, Viereck et al. identified another cardiac-specifically expressed lncRNA—cardiac hypertrophy-associated transcript (Chast)—as a candidate molecule that may influence myocardial remodeling. Chast was significantly upregulated in cardiomyocytes isolated from TAC-induced hypertrophic mice and patients with aortic stenosis. Subsequent *in vitro* and *in vivo* functional studies support the role of Chast as an endogenous prohypertrophic lncRNA (106).

The above studies emphasized the valuable diagnostic role of ncRNAs in the pathological myocardial remodeling. Meanwhile, understanding these entangled networks of RNA regulatory and functional interactions appeared to have promising implications in the characterization of the molecular events that control the progression of CVDs. Importantly, the manipulation of ncRNAs expression levels by either inhibiting or activating illustrates future novel therapeutic strategies in the pathological myocardial remodeling.

## RNA modification

Similar to DNA modification in epigenetics, accumulating evidence has implicated the presence of over one hundred types of RNA modification (107). RNA modification is an universal phenomenon of epigenetic regulation in eukaryotes, which endows RNA molecules with structural and functional diversity (108). One of the most common RNA modifications is methylation, which is indispensable in life and plays critical roles in biological functions. As key molecule linking DNA with protein, mRNA acts as a major carrier of genetic information, and thereby representing the most widely-studied populations in RNA methylation modification. In eukaryotic mRNA, different types of methylation modification have been documented, such as N^7^-methylguanosine (m^7^G), N^6^-methyladenosine (m^6^A), 5-methylcytosine (m^5^C), N^1^-methyladenosine (m^1^A), N4-acetylcytidine (ac^4^C), and 2'-O-methylation (Nm), with m^6^A being the most abundant modification in mRNA (Table 1).

**Table 1:** The roles of RNA modifications in CVDs.

**RNA modification**	**Related cardiovascular diseases**	**Levels**	**Key enzymes**	**Enzyme expression**	**Main functions**	**References**
m^6^A	Cardiac hypertrophy	Increased	METLL3	Upregulated	METLL3 induced remodeling in compensatory cardiac hypertrophy	(109)
	Atherosclerosis	Increased	METTL14	Upregulated	METTL14 promoted the proliferation and invasion of vascular endothelial cells	(110, 111)
	Ischemic heart disease	Increased	FTO	Downregulated	FTO overexpression improved cardiac function via inhibiting myocardial fibrosis and cellular apoptosis	(112)
m^1^A	Coronary heart disease	Decreased			A significantly lower level of m^1^A was detected in urines from CAD patients in comparison with those from non-CAD controls	(113)
m^7^G			METTL1		Depletion of METTL1 results in the upregulation of genes related to cardiovascular system development	(114, 115)
m^5^C	Atherosclerosis	Increased	NSUN2	Upregulated	NSun2 increased the adhesion of leukocytes to endothelial cells and contributed to the development of vascular endothelial inflammation and atherosclerosis	(116)
ac^4^C	Hypertension	Increased	NAT10	Upregulated	ac4C-mediated upregulation of inflammasome genes is closely involved in the pathogenesis of hypertension	(117)
Nm	Congenital heart disease	Decreased			scaRNAs-directed modification of Nm was able to regulating mRNA splicing variants which are crucial for heart development	(118)

m^6^A, N^6^-methyladenosine; m^1^A, N^1^-methyladenosine; m^7^G, N^7^-methylguanosine; m^5^C, 5-methylcytosine; ac4C, N^4^-acetylcytidine; Nm, 2'-O-methylation; METTL3, methyltransferase-like 3; METTL14, methyltransferase-like 14; FTO, fat mass and obesity-associated protein; CAD, coronary artery disease.

### N^6^-methyladenosine (m^6^A)

N^6^-methyladenosine (m^6^A) is generated by adding a methyl group at the N^6^ position of adenosine, which is highly conversed in sequence and ubiquitously found in most eukaryotes RNAs, such as yeast, plants, flies, mammals, and even viral RNAs, and plays an essential role in mRNA post-transcriptional modification and metabolism (119). m^6^A was originally detected in mammalian mRNAs in 1974 (120), then Jia et al. identified fat mass and obesity-associated protein (FTO) for the first time as m^6^A demethylase, implicating the dynamic and reversible nature of m^6^A methylation modification (121).

As a typical representative of epigenetic transcriptomics, m^6^A has profound effects on a series of RNA metabolism processes, including mRNA transcription, alternative splicing, transport, stability, degradation and miRNA processing (122), which are all confirmed to be closely involved in biological events such as stem cell renewal and differentiation, body growth and development, circadian rhythms, adipogenesis, spermatogenesis and carcinogenesis (123–125). Recently, m^6^A has been demonstrated to be catalyzed by a methyltransferase complex, which consists of Wilms' tumor 1-associating protein (WTAP), RNA-binding motif protein 15/15B (RBM15/15B), methyltransferase-like 3 (METTL3), and 14 (METTL14), and is demethylated by demethylases ALKB homo-log 5 (ALKBH5) and fat mass and obesity-associated protein (FTO) (126).

METTL3 and METTL14 are known as core components of the methyltransferase complex, with METTL3 functioning as a catalytic subunit, while METTL14 being responsible for substrate recognition, and they play a pivotal role in cardiovascular diseases. Dorn et al. have recorded that m^6^A methylation is increased in cardiomyocytes in response to hypertrophic stimulation, and enriched on genes involving protein kinases and intracellular signaling pathways. METTL3-mediated m^6^A methylation leads to compensated myocardial remodeling without interfering with cardiac function, whereas reduced m^6^A induced by METTL3 knockdown drives eccentric myocardial remodeling and dysfunction (109).

METTL3 was significantly upregulated in mouse model of myocardial infarction (MI) and TGF-β1-treated cardiac fibroblasts (CFs). *In vitro* and *in vivo* functional experiments showed that METTL3 overexpression can induce cardiac fibroblast proliferation and cardiac fibrosis, while knockdown of METTL3 exerted anti-fibrotic effects. Furthermore, RNA sequencing (RNA-seq) and m6A sequencing (m^6^A-seq) analyses revealed that METTL3 silencing caused an overall decrease of expression level and m^6^A level of fibrosis-related genes, implicating the pro-fibrotic property of METTL3 (127).

METTL3 was also found to be vital in maintaining the proliferative capacity of neonatal cardiomyocytes (128). Besides, METTL3 improved cardiomyocyte proliferation upon MI by upregulating miR-17-3p expression (129).

Interestingly, METTL14 has been reported to promote vascular endothelial cell proliferation and inflammation by increasing the m^6^A methylation modification of pre-miR-19a and forkhead box O1 (FOXO1), respectively, thus contributing to atherosclerosis. Both genetic ablation and siRNA-mediated knockdown of METTL4 can inhibit the development of atherosclerotic plaques (110, 111).

FTO is highly expressed in fetal heart ventricles and has been demonstrated by numerous studies to be closely involved in the pathogenesis of congenital heart disease [including hypertrophic cardiomyopathy (HCM), atrial septal defect (ASD) and ventricular septal defect (VSD)] (130), arrhythmias (131), coronary artery disease (CAD) (132), and metabolism-related heart disease (including obesity and type 2 diabetes) (133, 134). Mathiyalagan et al. utilized heart samples from human, pig and mouse as well as cultured cardiomyocytes to explore the potential role of m^6^A modification and FTO in failing hearts, and they concluded that FTO had diminished expression in mammalian failing hearts and hypoxia-induced cardiomyocyte models, which subsequently increases m^6^A RNA modification and impairs cardiomyocyte contractile function as a result, and that FTO overexpression in mouse models of MI can attenuate cardiac fibrosis and enhance angiogenesis, suggesting that FTO-dependent m^6^A methylation is of utmost importance in preserving cardiac function during heart failure (112).

### N^1^-methyladenosine (m^1^A)

N^1^-methyladenosine (m^1^A) was first extracted from rat liver by Dunn (135), and later identified as another naturally occurring RNA methylation modification manner different from m^6^A. Due to the nature of a positively charged methylated nucleoside (136), m^1^A markedly alter RNA structure and protein-RNA interactions through binding to translation initiation sites and the first splice site of mRNA under physiological conditions, and, especially, plays a critical role in promoting translation of methylated mRNA (137, 138). m^1^A is highly conversed in mammalian cells and is reversible by ALKBH1 and ALKBH3 in response to multiple cellular signal (139). A significantly lower level of m^6^A and m^1^A was detected in urines of CAD patients in comparison with those of non-CAD controls, indicating that methylated nucleoside may represent a novel and promising species of biomarkers for early diagnosis of CVD (113).

### N^7^-methylguanosine (m^7^G)

The 7-methylguanosine cap added to the 5' end of mRNA is essential for efficient gene expression and cell viability (140). N^7^-methylguanosine (m^7^G) modification is prevalent in all eukaryotic organisms in which it acts as a key regulator involved in different stages of gene expression, including transcription, mRNA splicing, nuclear export, translation and mRNA stability (141). Based on advanced high-throughput sequencing approaches developed for transcriptome-wide mapping of internal m^7^G modification, m^7^G DiseaseDB database is constructed and designated to identify the disease phenotypes that are most enriched with m^7^G variants. Among them, a considerable amount of variants are related to cardiovascular phenotypes, suggesting a strong linkage between m^7^G modification and CVD (142). Recently, a study have noted that METTL1-mediated m^7^G modification is necessary for self-renewal and differentiation of mouse embryonic stem cells (mESCs) and human induced pluripotent stem cells (hiPSCs), while depletion of METTL1 results in the upregulation of genes related to cardiovascular system development such as angiogenesis, thus implicating the potential of m^7^G modification as a therapeutic target in CVD therapy (114, 115).

### 5-methylcytosine (m^5^C)

The presence of 5-methylcytosine (m^5^C) in DNA and its role as an epigenetic regulator of genome activity has been well-established. In fact, m^5^C is also widely distributed in eukaryotic RNAs, mainly confined to tRNAs and rRNAs, as well as in mRNAs and other ncRNAs, and is involved in multiple cellular functions such as cell cycle control, cell proliferation and differentiation, and development (143). The important role of m^5^C post-transcriptional modification in mRNA metabolism focus mainly on the regulation of mRNA export and translation (144–146). As the major mRNA m^5^C methyltransferase, NSun2 upregulates the expression of ICAM-1 by methylating ICAM-1 mRNA, thereby increasing the adhesion of leukocytes to endothelial cells and contributing to the development of vascular endothelial inflammation and atherosclerosis (116).

### N4-acetylcytidine (Ac^4^C)

In contrast to other RNA modifications, N4-acetylcytidine (ac^4^C) remains poorly understood. Initially described in the bacterial tRNA^met^ anticodon several decades ago (147), ac^4^C was subsequently detected in eukaryotic tRNAs and 18S rRNA (148). More recently, Arango et al. documented for the first time the presence of ac^4^C modification in mRNAs, and they determined that mRNA acetylation within coding sequences can promote translation and mRNA stability (149). In all cases above, N-acetyltransferase 10 (NAT10) is the only known enzyme responsible for ac^4^C production (150). There has been robust evidence that ac^4^C-mediated upregulation of inflammasome genes, such as NLRC4 and NLRP3, is closely involved in the pathogenesis of cardiovascular disorders (151, 152). ac^4^C and adenine, when applied in combination, can activate the NLRC4 inflammasome, induce the IL-1β production in monocytes and neutrophils, enhance platelets and neutrophil activation as well as elevate blood pressure in mice (117). Moreover, the pro-inflammatory effect of NLRP3/IL-1β axis has been fully elucidated in the development of various CVDs, including atherosclerosis (153), atrial fibrillation (154), myocardial infarction (155) and ischemia-reperfusion injury (156), indicating that ac^4^C modification may be a promising therapeutic target in the future.

### 2'-O-methylation (Nm)

2'-O-methylation (Nm) is one of the most prevalent RNA modifications, which can occur within any ribonucleotide (Am, Cm, Gm, Um) (157, 158). Nm acts as an important epigenetic regulators of gene expression, which has recently been found to stabilize mRNA and inhibit its translation in mammalian cells (159, 160). To date, there has been accumulating evidence that Nm modification is strongly associated with CVD (161). According to the latest research, scaRNAs-directed modification of Nm is able to regulating mRNA splicing variants which are crucial for heart development, and disruption of which would alter regulatory processes leading to defective heart development and CHDs (118).

## Epigenetic-based therapies for pathological myocardial remodeling

The exploration and understanding of epigenetic landscapes during cardiometabolic disturbances furnish novel epigenetic targets that may become promising pharmacological interventions to mitigate the development of CVDs (162). Multiple preclinical studies have shown the cardioprotective effects of various epigenetic-based therapies (“epidrugs”), including pharmacological DNA methylation inhibitors, histone deacetylase inhibitors (HDACi), BET inhibitors, and so on (163) (Table 2).

**Table 2:** The multiple cardiac diseases and their corresponding epigenetic pathogenesis.

**Diseases**	**Species**	**Epigenetic regulation**	**Epigenetic pathogenesis**	**References**
Cardiac remodeling	Mice	Histone acetylation and deacetylation	HDAC2 overexpression	(24)
	Mice	Histone acetylation and deacetylation	Cardiac-specific HDAC3-deficient	(25)
	Mice	Histone acetylation and deacetylation	MEF2 activity increased	(27)
	Mice	Histone methylation and demethylation	H3K4me3 changed in the promoter of fetal heart genes	(57)
	Mice	Histone methylation and demethylation	JMJD2A activation	(59)
	Mice	Histone ribosylation	PARP-1 activity increased	(65)
	Human	Histone acetylation and deacetylation	SIRT3 activity decreased	(52)
	Mice	ATP-dependent chromatin remodeling	Cardiac-specific conditional knockout of the HIRA	(87)
	Human/Mice	miRNAs	Downregulated miR1/133; miR21/22 overexpression	(91–94)
	Human	lncRNA	LIPCAR increased	(102)
	Mice	lncRNA	H19 increased	(103)
	Mice	lncRNA	Mhrt expression diminished	(105)
	Human	lncRNA	Chast increased	(106)
	Mice	m^6^A	METTL3 knockdown	(109)
Heart failure	Human	DNA methylation	Hypomethylated CGI and promoters	(7)
	Rat	Histone acetylation and deacetylation	Acetylation or activation of HATs	(27–29)
	Human	Histone acetylation and deacetylation	SIRT1 activity decreased	(42, 44)
	Mice	Histone methylation and demethylation	Conditional knockout of G9a	(58)
	Mice	Histone ribosylation	PARP-1 activity increased	(65)
	Human	ATP-dependent chromatin remodeling	BAF complex activity increased	(83)
	Human	miRNAs	miR765 overexpression	(95)
	Human	lncRNA	LIPCAR increased	(102)
	Human/Mice	m^6^A	FTO knockdown	(112)
Dilated	Human	DNA methylation	CpG methylation increased	(12)
cardiomyopathy	Mice	Histone acetylation and deacetylation	Cardiac-specific deletion of HDAC1 or HDAC2	(23)
	Mice	Histone acetylation and deacetylation	Cardiac-specific overexpression of p300	(32)
	Mice	miRNAs	miR340 overexpression	(97)
Congenital heart defects	Mice	Histone acetylation and deacetylation	Loss of HDAC1/2	(23)
Atherosclerosis	Mice	Histone acetylation and deacetylation	SIRT1 activity decreased	(38, 39)
	Human	m^6^A	METTL4 increased	(110)
	Human	m^5^C	NSun2 increased	(116)
Myocardial	Human	Histone acetylation and deacetylation	SIRT1 activity decreased	(43)
infarction	Rat	m^6^A	METTL3 knockdown	(129)
Hypertension	Human	Histone acetylation and deacetylation	SIRT3 activity decreased	(48)

HATs, histone acetyltransferases; HDACs, histone deacetylases; MEF2, myocyte enhancer factor 2; PARP-1, poly (ADP-ribose) polymerase-1; H3K4me3, histone 3 lysine 4 triamcinolone; SIRT1, Sirtuins 1; Mhrt, myosin heavy-chain-associated RNA transcripts; Chast, cardiac hypertrophy-associated transcript; miRNAs, microRNAs; lncRNAs, long ncRNAs; m^6^A, N^6^-methyladenosine; m^5^C, 5-methylcytosine; METTL3, methyltransferase-like 3; FTO, Fat mass and obesity-associated protein.

### DNA methylation inhibitors

The reversible methylation status and expression levels of target genes offers researchers a promising prospect for developing the potential effects of DNA methyltransferase inhibitors or related epidrugs on improving pathological myocardial remodeling. Hydrazine, a drug originally designed to lower blood pressure, was found to maintain Ca (2+) homeostasis and improve cardiac function by enhancing myocardial sarcoplasmic reticulum Ca (2+)-ATPase (SERCA2a) activity and inducing DNA demethylation in the gene promoter in cardiomyocytes (164). The supplementation of selenium which is an essential trace element showed obvious cardioprotective effects toward advanced glycation end products (AGEs)-induced heart failure via inhibiting DNMT2-induced DNA methylation of glutathione peroxidase 1 (GPX1) gene promoter in myocytes (165). Moreover, as an inhibitor of DNA methylation, 5-Azacytidine (5aza) has been shown to be effective in attenuating pressure overload-induced myocardial remodeling (166).

### HDACi

HDACi, which exerted anti-proliferative and anti-inflammatory effects, are the largest class of epidrugs that may be highly correlated with the prevention and treatment of CVDs (167). Indeed, HDACs could modulate acetylation status of histone proteins. The current preclinical studies indicated that HDACi could mitigate cardiac pathological remodeling and improve myocardial function via acetylating or deacetylating target genes (168, 169). The available HDACi, suberoylanilide hydroxamic acid (SAHA), was reported to attenuate ischemia/reperfusion-induced cardiac injure through the induction of cardiomyocyte autophagic flux (170). Also, sodium butyrate has been identified to blunt myocardial I/R injury and preserve cardiac performance by inhibiting the activity of histone deacetylases (171, 172). Aliphatic acids like valproic acid (VPA), an inhibitor of class I HDACs, has been reported to attenuate MI-induced myocardial remodeling by inhibiting the activities of histone deacetylases and subsequently accumulating hyperacetylated histone tails (173). Entinostat (MS-275A), another inhibitor of class I HDACs, has also been reported to play a protective role in ischemia reperfusion injury-induced myocardial remodeling (174). Besides, the class II HDACi could prevent myocardial hypertrophy and cardiac fibrosis (175). However, Trichostatin A (TSA), an inhibitor of both class I and II HDACs, showed some severe side effects including DNA damage, which limited its clinical use (176).

### BET inhibitors

The effects of some compounds (i.e., folates, apicidin, and valproic acid) on regulating chromatin accessibility that changed in cardiometabolic disease states by modulating epigenetic marks on DNA or histones aroused the interest of developing them as CVD-related epidrugs (177, 178). Apabetalone (RVX-208), a direct epidrug, was shown to mitigate the development of CVDs and reduce major adverse cardiovascular event (MACE) risk by binding to the BET family member BRD4 (179, 180). The transcriptional regulation of RVX-208 targeting BRD4 could modulate cholesterol metabolism and vascular inflammation, contributing to its effects on attenuating cardiac hypertrophy and fibrosis (181, 182). Also, in mice or cultured cells, the small-molecule inhibitor JQ1 (a small molecular inhibitor of BET acetylycine reader protein) could inhibit norepinephrine-induced cell hypertrophy. It has also been reported that the use of JQ1 had therapeutic effects on animal models of pathological myocardial remodeling (183).

### Exosome non-coding RNAs

The rapid development of the nucleotide gene therapies which includes antisense oligonucleotide (ASO) and siRNA may usher in the potential breakthrough of the treatment targeting non-coding RNAs in ameliorating the symptoms and prognosis of patients with CVDs (184, 185). Notably, the easy-synthesized mimic or analogs or inhibitors of non-coding RNA are considered low toxic when transfected *in vivo* (184), which may pave the way for the use of exosome miRNAs and/or lncRNAs as new tools for diagnosing and treating pathological myocardial remodeling.

### Molecules targeting RNA modification

Multiple potent and selective small molecule compounds targeting RNA modification have been confirmed and applied mainly in cancer-related fields (186). Recently, a few activators or inhibitors of m6A enzymes including METTL3–METTL14 complex have also been developed (187, 188). Remodelin, a small molecule inhibitor of NAT10, was reported to alleviate cancers or inflammatory diseases (189, 190). However, the effectiveness of these compounds in CVDs remain to be demonstrated in the future. Moreover, epitranscriptome editing, a novel genome editing tool, could modify m6A via editing mutated or dysregulated functional m6A sites, making it worthy of being a promising approach to the clinical treatment of pathological myocardial remodeling subsequent to heart failure (191).

However, despite a variety of experimental evidence demonstrated above, the degree to which these preclinical observations or findings could be translated into humans is still relatively unknown, which were looking forward to being tested in more standardized clinical trials.

### The repurposed drugs

The repurposed drugs with a possible indirect epigenetic interference, including metformin, statins, sodium glucose transporter inhibitors 2 (SGLT2i), and omega 3 polyunsaturated fatty acids (PUFAs), have been identified by a number of preclinical and clinical experiments as having the cardioprotective effects in cardiac cells (163, 192). Specifically, it was reported that metformin could ameliorate cardiac fibrosis (193). Metformin have epigenetic-oriented effects by inhibiting the activity and expression of multiple histone methyltransferases, HATs, and DNMTs, thus affecting the transcription of target genes implicated in some vital signaling pathways (194). Statins may also participate in the prevention and treatment of CVDs through histone modifications (195). There are still few randomized trials have been carried out to evaluate whether the potential epigenetic-oriented effects of these non-canonical repurposed drugs, which were not originally developed as epidrugs, were involved in their cardioprotective effects against different clinical phenotypes of heart failure (163).

### Prospects and challenges

The detrimental effects of epigenetically altered gene expression profiles elicited by undesirable environmental conditions are increasingly posing an overwhelming threat to human health status, leading to higher prevalence of CVD worldwide. In this mini-review, we summarize the current knowledge about mechanistic basis for epigenetic regulation, which includes DNA methylation, histone modifications, ATP-dependent chromatin structural remodeling, non-coding RNAs and RNA modification, and further discuss the potential role of epigenetic regulation in pathological myocardial remodeling.

Despite advancements in this new realm, however, there still exist some restrictions that hamper the therapeutic exploitation of epigenetic regulators in clinical applications.

- First to be noted, most systemic epigenetic regulators possess limited cardiac-specificity, and instead, they alter gene expression in a relatively routine and comprehensive manner, which may result in an array of adverse effects.
- Second, due to the complexity of cell type composition in cardiac tissue and cell type-specific epigenetic characteristics in disease progression, studies in which changes in epigenetic profile of each cellular component in hypertrophic heart are still lacking. Pursuing cell-specific epigenetic information that changes in the cardiometabolic processes may advance individualized risk assessment and individualized pharmacotherapies for patients.
- Third, the establishment and refinement of appropriate targeted therapeutic strategies with high effectiveness and safety and low incidence of off-target mutations based on epigenetic mechanisms remain a great challenge to date.
- Forth, more research is needed to acquire a comprehensive understanding of how epigenetic regulation perform on cardiovascular disease so as to open up new avenues for pathological myocardial remodeling therapy.
- Finally, precisely defined patient cohorts in whom one pathomechanism is the sole or dominant cause of disease development and progression as well as defined selection criteria and suitable clinical outcome parameters needs to be highly considered in future clinical applications.

In the near future, with the tremendous changes in disease spectrum, rapid emergence of advanced sequencing technologies and increasing demand for novel pathological myocardial remodeling therapy, these gaps could be partly overcome using innovative bioinformatic tools designed to disentangle the epigenetic landscape of myocardial remodeling in man, which may provide improved precision medicine and personalized therapeutic approaches for patients at high risk of myocardial remodeling. In parallel, the continuous research and development on the molecular mechanisms underlying the cardioprotective effects of epidrugs would definitely broaden their application prospects for beneficial to patients with pathological myocardial remodeling or cardiometabolic disturbances.

## Author contributions

All authors listed have made a substantial, direct, and intellectual contribution to the work and approved it for publication.

## Conflict of interest

The authors declare that the research was conducted in the absence of any commercial or financial relationships that could be construed as a potential conflict of interest.

## Publisher's note

All claims expressed in this article are solely those of the authors and do not necessarily represent those of their affiliated organizations, or those of the publisher, the editors and the reviewers. Any product that may be evaluated in this article, or claim that may be made by its manufacturer, is not guaranteed or endorsed by the publisher.

## Glossary

5mC: 5th carbon atom
CGI: CpG islands
MeDIP: Methylated DNA immunoprecipitation
DCM: Dilated cardiomyopathy
LY75: Lymphocyte antigen 75
ERBB3: Tyrosine kinase-type cell surface receptor HER3
HOXB13: Homeobox B13
ADORA2: Aadenosine receptor A2A
HATs: Histone acetyltransferases
HDACs: Histone deacetylation enzymes
MEF2: Myocyte enhancer factor 2
CaMKII: Calcium/calmodulin-dependent protein kinase II
PKA: Protein kinase A
CBPCREB: Binding protein
MI: Myocardial infarction
H3K4me3: Histone 3 lysine 4 triamcinolone
β-MHC: Myosin heavy chain
HMT: Histone methyl transferase
TAC: Aortic narrowing surgery
HDM: Histone trimethyldemethylase
FHL1: Four and a Half LIM Domains Protein 1
PTIP: PAX transcriptional activation domain interaction protein
PARP-1: Poly (ADP-ribose) polymerase-1
NAD +: Nicotinamide adenine dinucleotide
BET: Bromine domain/BCD domain
TSS: Transcription start site
ANF: Atrial natriuretic factors
ADCR: ATP-dependent chromatin remodeling complex
ATPase: Adenosine triphosphate
MHC: Myosin heavy chain
DMNT3: DNA methyltransferase
ncRNAs: Non-coding RNAs
tRNAs: Transfer RNAs
rRNAs: Ribosomal RNAs
miRNAs: MicroRNAs
siRNAs: Small interfering RNA
snRNAs: Small nuclear RNAs
snoRNAs: Small nucleolar RNAs
scRNAs: Small cytoplasmic RNAs
exRNAs: Extracellular RNAs
piRNAs: Piwi-interacting RNAs
lncRNAs: Long ncRNAs
CVD: Cardiovascular disease
mRNAs: Messenger RNAs
TAC: Transverse aortic constriction
RasGAP: Ras GTPase-activating protein
Cdk9: Cyclin-dependent kinase 9
Rheb: Ras homolog enriched in brain
Chaer: Cardiac-hypertrophy-associated epigenetic regulator
PRC2: Polycomb repressor complex 2
H3K27me3: Histone H3 lysine 27 methylation
hiPSC-CMs: Pluripotent stem cell-derived cardiomyocytes
Mhrt: Myosin heavy-chain-associated RNA transcripts
Chast: Cardiac hypertrophy-associated transcript
m^7^G: N^7^-methylguanosine
m^6^A: N^6^-methyladenosine
m^5^C: 5-methylcytosine
m^1^A: N^1^-methyladenosine
ac^4^C: N4-acetylcytidine
Nm: 2'-O-methylation
FTO: Fat mass and obesity-associated protein
WTAP: Wilms' tumor 1-associating protein
RBM15/15B: RNA-binding motif protein 15/15B
METTL3: Methyltransferase-like 3
METTL14: Methyltransferase-like 14
ALKBH5: ALKB homo-log 5
CFs: Cardiac fibroblasts
RNA-seq: RNA sequencing
m^6^A-seq: m6A sequencing
FOXO1: Forkhead box O1
HCM: Hypertrophic cardiomyopathy
ASD: Atrial septal defect
VSD: Ventricular septal defect
CAD: Coronary artery disease
m^1^A: N^1^-methyladenosine
m^7^G: N^7^-methylguanosine
mESCs: Mouse embryonic stem cells
hiPSCs: Human induced pluripotent stem cells
m^5^C: 5-methylcytosine
ac^4^C: N4-acetylcytidine
NAT10: N-acetyltransferase 10
Nm: 2'-O-methylation.
